# WHO Statement on Caesarean Section Rates

**DOI:** 10.1111/1471-0528.13526

**Published:** 2015-07-22

**Authors:** AP Betran, MR Torloni, JJ Zhang, AM Gülmezoglu, the WHO Working Group on Caesarean Section, HA Aleem, F Althabe, T Bergholt, L de Bernis, G Carroli, C Deneux‐Tharaux, R Devlieger, S Debonnet, T Duan, C Hanson, J Hofmeyr, R Gonzalez Pérez, A de Jonge, K Khan, S Lansky, G Lazdane, P Lumbiganon, D Mackeen, R Mahaini, S Manyame, M Mathai, R Mikolajczyk, R Mori, B De Mucio, OT Oladapo, E Ortiz‐Panozo, L Ouedraogo, C Parker, M Robson, S Serruya, JP Souza, CY Spong, C Stanton, ME Stanton, EA Sullivan, M Temmerman, A Tita, Ӧ Tunçalp, P Velebil, JP Vogel, M Weber, D Wojdyla, J Ye, K Yunis, J Zamora, A Zongo

**Affiliations:** ^1^ UNDP, UNFPA, UNICEF, WHO, World Bank Special Programme of ResearchDevelopment and Research Training in Human ReproductionDepartment of Reproductive Health and ResearchWorld Health OrganizationGenevaSwitzerland; ^2^ Brazilian Cochrane Center and Department of ObstetricsSão Paulo School of MedicineSão Paulo Federal UniversitySão PauloBrazil; ^3^ Ministry of Education–Shanghai Key Laboratory of Children's Environmental HealthXinhua HospitalShanghai Jiao Tong University School of MedicineShanghaiChina

In 1985 when a group of experts convened by the World Health Organization in Fortaleza, Brazil, met to discuss the appropriate technology for birth, they echoed what at that moment was considered an unjustified and remarkable increase of caesarean section (CS) rates worldwide.^1^ Based on the evidence available at that time, the experts in Fortaleza concluded: ‘there is no justification for any region to have a caesarean section rate higher than 10–15%’.^1^ Over the years, this quote has become ubiquitous in scientific literature, being interpreted as the ideal CS rate. Although this reference range was intended for ‘populations’, which are defined by geopolitical boundaries, in many instances it has been mistakenly used as the measurement for healthcare facilities regardless of their complexity or other characteristics. In addition to the case mix of the obstetric population served, the use of CS at healthcare facilities is also affected by factors such as their capacity to handle cases, availability of resource and the clinical management protocols used locally.

Since its publication and for the last 30 years, this reference rate for CS has received intense criticism and has led to controversy, concern, polarised opinions and heated debates, while in parallel, the use of CS as a mode of delivery has continued its worrying rise worldwide. The need to revisit the recommended CS rate has been considered more and more necessary in view of the significant improvements in clinical obstetric care and in the methodology to assess evidence and issue recommendations in the last three decades.

The global concern around CS rates is understandable. When medically justified, a CS can prevent maternal and perinatal mortality and morbidity. There is no evidence, however, showing the benefits of the procedure for women or infants where it is not required. CS is associated with short‐ and long‐term risk, which can extend beyond the current delivery and affect future pregnancies. In addition, the increase in CS rates seems uncontrollable, with no signs that it is slowing down. The situation is aggravated by the fact that the causes of the rise are not fully understood but emerge as a complex multifactorial labyrinth involving health systems, health care providers, women, societies, and even fashion and media.^2^, ^3^, ^4^, ^5^, ^6^ Lastly, non‐clinical interventions to reduce unnecessary CS have shown limited effectiveness to date.^7^

In light of these issues, WHO convened a meeting in Geneva, Switzerland, on 8–9 October 2014 with the objective of (1) establishing the current WHO position on the CS rate or range for optimal maternal and perinatal outcomes at population level, and (2) agreeing on a proposal for a tool to monitor CS rates at facility level. The *Statement on Caesarean Section Rates* recently released by WHO summarises the results of the systematic reviews and analyses conducted for this purpose and conveys the thinking emerging from the discussions of the meeting.^8^

A systematic review and an ecological analysis were performed and concluded that at population level, CS rates higher than 10% were not associated with reductions in maternal and newborn mortality rates.^9^, ^10^ The Statement notes, however, that the association between CS rates and other relevant outcomes such as stillbirths, maternal and perinatal morbidity, paediatric outcomes and psychological or social well‐being could not be determined due to the lack of data on these other outcomes at the population level. This lack of data represents a limitation of these analyses that needs to be borne in mind.

Beyond numbers and rates, the Statement emphasises that the critical role played by the quality of care in this equation cannot be overstated. As with any surgery, CS is associated with short‐ and long‐term risks, particularly in settings that lack the facilities or capacity to conduct safe surgery or treat surgical complications properly, or where access to labour care or repeat CS in subsequent pregnancies cannot be taken for granted. On the other hand, inadequate access to timely CS may result in perinatal asphyxia, stillbirth, uterine rupture or obstetric fistula, a marker for exceptionally prolonged, obstructed labour.^11^ Thus, CS should be undertaken when medically necessary, and rather than striving to achieve a specific rate, efforts should focus on providing caesarean section to all women in need. How to define the woman ‘in need’ can only be ascertained by the health care providers caring for the woman on a case‐by‐case basis.

Most importantly, at the healthcare facility level, clinicians and administrators struggle to monitor CS rates in a meaningful, reliable and action‐oriented manner. Historically, caesarean sections have often been categorised using its indications as the unit being classified. Using indications to classify CS has always been problematic due to the lack of uniform definitions for most common indications and has resulted in poor reproducibility and unsatisfactory comparisons.^12^ In 2001, Dr Michael Robson proposed a system of 10 groups that classifies all women admitted for delivery (and not indications) according to five obstetric characteristics that are generally routinely collected in most maternities.^13^ Two systematic reviews conducted at WHO identified this classification as the most appropriate system to fulfil current international and local needs.^12^, ^14^ The WHO Statement proposes the use of the Robson classification as the global standard for assessing, monitoring and comparing CS rates within healthcare facilities over time, and between facilities. In the last decade, this classification has witnessed an extraordinary expansion in its use worldwide, particularly in healthcare facilities, due to its intrinsic appealing characteristics: simplicity of design, validity of purpose, ease of implementation and directness of initial interpretation.^14^ WHO envisions that the information stemming from the classification can be a powerful tool to inform practice. The classification will allow not only for stratification of CS rates in more uniform groups of women but also the assessment of CS rates in relation to other perinatal outcomes and processes (e.g. rates of oxytocin usage, postpartum haemorrhage, newborn outcomes, length of labour).

WHO will guide and support countries in the use, implementation and interpretation of the classification so that we can start comparing CS rates in a meaningful, targeted, transparent and useful manner. By endorsing the Robson classification, this Statement should become a catalyst for action. The time has come to put the debate about the preferable rate of CS on hold. Let's start to collect data uniformly so that in the near future we will be able to move our focus from CS rates at population level to monitoring and discussing CS rates and outcomes in each group of the Robson classification. Only then will we have the data and evidence that will lead us more clearly to actions to improve care.^15^ Ultimately, we hope the debate can recommence with more valuable, solid and informative data to support our discussions.

## Disclosure of interests

None declared. Completed disclosure of interests form available to view online as supporting information.

## Contribution to authorship

All authors contributed to the writing of the commentary.

## Funding

This commentary has been written without any external funding.

## Details of ethics approval

No ethical approval was sought for the writing of this commentary.

## Disclaimer

The authors alone are responsible for the views expressed in this publication and they do not necessarily represent the decisions or policies of the World Health Organization or other organizations.

## Appendix

WHO Working Group on Caesarean Section: HA Aleem (Department of Obstetrics and Gynecology, Women's Health Center, Assiut University Hospital, Assiut, Egypt), F Althabe (Institute for Clinical Effectiveness and Health Policy, Buenos Aires, Argentina), T Bergholt (Department of Obstetrics, University of Copenhagen, Copenhagen, Denmark), L de Bernis (United Nations Population Fund, Geneva, Switzerland), G Carroli (Centro Rosarino de Estudios Perinatales, Rosario, Argentina), C Deneux‐Tharaux (INSERM U1153, Obstetrical, Perinatal and Pediatric Epidemiology Research Team, Center for Epidemiology and Statistics Sorbonne Paris Cité, Paris Descartes University, Paris, France), R Devlieger (UZLeuven, Campus Gasthuisberg, Department of Obstetrics and Gynecology, Leuven, Belgium), S Debonnet (International Confederation of Midwives, 2517 AN The Hague, the Netherlands), T Duan (Shanghai No.1 Maternal & Infant Health Hospital, Shanghai, China), C Hanson [International Federation of Gynecology & Obstetrics (FIGO), London, UK], J Hofmeyr (Department of Health, Effective Care Research Unit, University of Fort Hare, East London, Eastern Cape, South Africa), R Gonzalez Pérez (Department of Maternal and Gynaecological Health, Pontificia Universidad Catolica de Chile, Santiago de Chile, Chile), A de Jonge (Midwifery Science, AVAG and the EMGO Institute of Health and Care Research, VU University Medical Center, Amsterdam, the Netherlands), K Khan (Women's Health Research Unit, Multi‐disciplinary Evidence Synthesis Hub, The Blizard Institute, London, UK), S Lansky (Ministry of Health, Belo Horizonte Minas Gerais, Brazil), G Lazdane (WHO Regional Office for Europe, Copenhagen, Denmark), P Lumbiganon (Department of Obstetrics and Gynecology, Faculty of Medicine, Khon Kaen University, Khon Kaen, Thailand), D Mackeen (Department of Obstetrics and Gynecology, Division of Maternal–Fetal Medicine, Geisimger Health System, Danville, PA, USA), R Mahaini (WHO Office for the Eastern Mediterranean Region, Cairo, Egypt), S Manyame (Department of Obstetrics & Gynaecology, Harare Hospital & University of Zimbabwe, Harare, Zimbabwe), M Mathai (Department of Maternal and Child Health, World Health Organization, Geneva, Switzerland), R Mikolajczyk (ESME – Epidemiological and Statistical Methods Research Group, Helmholtz Centre for Infection Research, Braunschweig, Germany), R Mori (Department of Health Policy, National Center for Child Health and Development, Tokyo, Japan), B De Mucio (Latin American Center for Perinatology, Women and Reproductive Health (CLAP/WR), WHO Regional Office for the Americas, Montevideo, Uruguay), OT Oladapo (UNDP‐UNFPA‐UNICEF‐WHO‐World Bank Special Programme of Research, Development and Research Training in Human Reproduction, Department of Reproductive Health and Research, WHO, Geneva, Switzerland), E Ortiz‐Panozo (Center for Population Health Research, National Institute of Public Health, Cuernavaca, Mexico), L Ouedraogo (WHO Regional Office for Africa, Brazzaville, Congo), C Parker (Obstetrics and Gyneacology Department, Baragwanath Maternity Hospital, Johannesburg, South Africa), M Robson (National Maternity Hospital, Dublin, Ireland), S Serruya (Latin American Center for Perinatology, Women and Reproductive Health (CLAP/WR), WHO Regional Office for the Americas, Montevideo, Uruguay), JP Souza [Department of Social Medicine, Ribeirão Preto Medical School, University of São Paulo, Ribeirão Preto (SP), Brazil], CY Spong (Deputy Director, *Eunice Kennedy Shriver* National Institute of Child Health and Human Development National Institutes of Health, Bethesda, MD, USA), C Stanton (Johns Hopkins Bloomberg School of Public Health, Baltimore, MD, USA), ME Stanton (USAID, Washington DC, USA), EA Sullivan (Faculty of Health, University of Technology, Sydney, Australia), M Temmerman (UNDP‐UNFPA‐UNICEF‐WHO‐World Bank Special Programme of Research, Development and Research Training in Human Reproduction, Department of Reproductive Health and Research, WHO, Geneva, Switzerland), A Tita (Department of Obstetrics and Gynecology, Division of Maternal–Fetal Medicine, University of Alabama at Birmingham, Birmingham, AL, USA), Ӧ Tunçalp (UNDP‐UNFPA‐UNICEF‐WHO‐World Bank Special Programme of Research, Development and Research Training in Human Reproduction, Department of Reproductive Health and Research, WHO, Geneva, Switzerland), P Velebil (Perinatal Center of the Institute for the Care of Mother and Child, Prague, Czech Republic), JP Vogel (UNDP‐UNFPA‐UNICEF‐WHO‐World Bank Special Programme of Research, Development and Research Training in Human Reproduction, Department of Reproductive Health and Research, WHO, Geneva, Switzerland), M Weber (WHO Regional Office for Europe, Copenhagen, Denmark), D Wojdyla (Duke Clinical Research Institute, Durham, NC, USA), J Ye (Ministry of Education–Shanghai Key Laboratory of Children's Environmental Health, Xinhua Hospital, Shanghai Jiao Tong University School of Medicine, Shanghai, China), K Yunis (Department of Pediatrics & Adolescent, Medicine and Department of Pediatrics, American University of Beirut, Beirut, Lebanon), J Zamora (Clinical Biostatistics Unit, Hospital Ramón y Cajal, Madrid, Spain), A Zongo (Research Institute for Development, Université Paris Descartes, Sorbonne Paris Cité, UMR 216, Paris, France and Direction de la santé de la famille, Ministère de la Santé, Ouagadougou, Burkina Faso)
